# Community-based breeding programs can realize sustainable genetic gain and economic benefits in tropical dairy cattle systems

**DOI:** 10.3389/fgene.2024.1106709

**Published:** 2024-05-16

**Authors:** Direba Hunde, Yosef Tadesse, Million Tadesse, Solomon Abegaz, Tesfaye Getachew

**Affiliations:** ^1^ Ethiopian Institute of Agricultural Research, Holetta Center, Holetta, Ethiopia; ^2^Department of Animal Science, Haramaya University, Harar, Ethiopia; ^3^ Woldia University, Woldia, Ethiopia; ^4^ International Center for Agricultural Research in the Dry Areas, Addis Ababa, Ethiopia

**Keywords:** accuracy, community-based breeding, discounted profit, generation interval, genetic gain, smallholder

## Abstract

Implementing an appropriate breeding program is crucial to control fluctuation in performance, enhance adaptation, and further improve the crossbred population of dairy cattle. Five alternative breeding programs (BPs) were modeled considering available breeding units in the study area, the existing crossbreeding practices, and the future prospects of dairy research and development in Ethiopia. The study targeted 143,576 crossbred cows of 54,822 smallholder households in the Arsi, West Shewa, and North Shewa zones of the Oromia Region, as well as the North Shewa zone of the Amhara Region. The alternative BPs include conventional on-station progeny testing (SPT), conventional on-farm progeny testing (FPT), conventional on-station and on-farm progeny testing (SFPT), genomic selection (GS), and genomic progeny testing (GPT). Input parameters for modeling the BPs were taken from the analysis of long-term data obtained from the Holetta Agricultural Research Center and a survey conducted in the study area. ZPLAN+ software was used to predict estimates of genetic gain (GG) and discounted profit for goal traits. The predicted genetic gains (GGs) for milk yield (MY) per year were 34.52 kg, 49.63 kg, 29.35 kg, 76.16 kg, and 77.51 kg for SPT, FPT, SFPT, GS, and GPT, respectively. The GGs of the other goal traits range from 0.69 to 1.19 days per year for age at first calving, from 1.20 to 2.35 days per year for calving interval, and from 0.06 to 0.12 days per year for herd life. Compared to conventional BPs, genomic systems (GPT and GS) enhanced the GG of MY by 53%–164%, reduced generation interval by up to 21%, and improved the accuracy of test bull selection from 0.33 to 0.43. The discounted profit of the BPs varied from 249.58 Ethiopian Birr (ETB, 1 USD = 39.55696 ETB) per year in SPT to 689.79 ETB per year in GS. Genomic selection outperforms SPT, SFPT, and FPT by 266, 227%, and 138% of discounted profit, respectively. Community-based crossbreeding accompanied by GS and gradual support with progeny testing (GPT) is recommended as the main way forward to attain better genetic progress in dairy farms in Ethiopia and similar scenarios in other tropical countries.

## 1 Introduction

Ethiopia has a large cattle population with an estimated 66.26 million head (CSA, 2022). Most (97%) of these cattle are indigenous. This report revealed that the milk yield performance of indigenous cows is generally low (1.45 kg per day). As a result, crossbreeding has been practiced as a novel intervention for the development of dairy cattle in central Ethiopia. The performance and economic contributions of crossbred dairy cattle for smallholder farmers were substantial (Aynalem et al., 2011; Kefena et al., 2013; Direba et al., 2022). They noted that crossbred dairy cows perform better than indigenous cows by 3 to 7 folds of milk yield per lactation. Crossbred cows attain their age at first calving (35–37 months) earlier than indigenous cows (42–60 months) in Ethiopia. Smallholder farmers with crossbred dairy cattle generate significantly higher income (40%) than those who keep only indigenous cattle (Agajie et al., 2016).

The better results of crossbred dairy cattle than indigenous cattle are mainly due to the additive gene contribution of dairy breeds and the heterosis effect (Direba et al., 2022). However, continued up-grading toward exotic dairy breeds resulted in an adaptive problem. Loss of heterosis in the inter-se generations of crossbred cattle led to a decline in performance (Gradiz et al., 2009; Hatungumukama and Detilleux, 2009; Aynalem et al., 2011). For instance, the milk yields of F2 and F3 50% Friesian × Boran cows decreased by 26% and 30%, respectively, compared to F1 cows (Direba et al., 2022). Several countries have practiced breeding programs that resulted in noticeable genetic progress toward the breeding goal traits of dairy cattle (Weller et al., 2017; Van Marle-Köster and Visser, 2018). According to Weller et al. (2017), the mean annual milk production of dairy cows has increased from 7,000 kg to 13,000 kg per cow since the 1970s in Israel. A few crossbreeding programs have been implemented in tropical countries, including Ethiopia at the on-station level (research institutes), to tackle the problems associated with adaptation and decreased performance in the next generations. However, no substantial improvement or sustainable breeding program has been implemented for crossbred dairy cattle at the smallholder level in Ethiopia. Thus, studies and strategic documents have recommended designing and implementing appropriate breeding programs to control fluctuation in performance, enhance adaptation, and further improve the population (FAO, 2010; Philipsson et al., 2010; EIAR, 2017; MOA, 2019). There are large crossbred cattle populations (2 million), sufficient genetic variance, and medium-to-good heritability for breeding goal traits to improve crossbred dairy cattle through selection in Ethiopia (Gebregziabher et al., 2013; CSA, 2017; Direba et al., 2022). The objective of this study was to evaluate alternatives and develop an appropriate breeding program for crossbred dairy cattle of smallholder farmers in central Ethiopia.

## 2 Materials and methods

### 2.1 Study area

The study area for simulating alternative breeding programs (BPs) focused on smallholder farmers owning crossbred cattle in specific zones of the Oromia Region (Arsi, West Shewa, and North Shewa) and the North Shewa zone of the Amhara Region, Ethiopia. These areas are home to a significant cattle population, estimated at 7,958,831 head [2,253,959 in West Shewa, 1,676,748 in north shewa zone of oromia region (NSHORO), 2,545,778 in Arsi, and 1,482,346 in north shewa zone of Amhara region (NSHAMA)] according to CSA (2017). Crossbreeding practices have been widely adopted in these areas over the past 4 to 5 decades, resulting in approximately 377,729 crossbred cattle, accounting for 35.2% of the national crossbred cattle population (CSA, 2017). The geographical proximity of the study areas, within a 150 km radius of the capital city, Addis Ababa, facilitates the supply of inputs and outputs. For further details on the study area’s geographic references and climate information, refer to Direba et al. (2020).

### 2.2 Simulation of alternative breeding programs

The simulation of alternative breeding programs (BPs) involved considering genetic parameters, economic values of breeding goal traits, biological and technical parameters, and cost parameters. The goal traits included lactation milk yield (MY), age at first calving (AFC), calving interval (CI), and herd life (HL). Five different BPs were designed and compared within the study area while taking into account the available breeding unit at Holeta Research Center, existing crossbreeding practices, and future prospects of dairy research and development in Ethiopia. These breeding programs included conventional on-station progeny testing (SPT), conventional on-farm progeny testing (FPT), conventional on-station and on-farm progeny testing (SFPT), genomic selection (GS), and genomic progeny testing (GPT). The first three conventional BPs were simulated without utilizing genomic information. A total of 143,576 crossbred cows were assumed to model the alternative BPs. The data collection and selection were presumed only in the breeding unit (BU), and no data were collected from cattle in production units (PUs) in all BPs.

In the FPT, SFPT, GS, and GPT breeding programs, smallholder farmers are expected to participate by providing cows for progeny testing, allowing selected cows or male calves for genotyping, selling the selected male calves to the breeding program, and maintaining records. An on-farm survey indicated that smallholder farmers own an average of 2.65 cows (Direba et al*.,* 2020); approximately 1,000 farmers would participate in implementing the progeny testing scheme. Additionally, around 25 to 27 data recorders should be employed to gather data from the cows in a breeding unit. Over time, the responsibility of data recording in the BU can be transferred to the cattle owners through training and creating awareness about the potential advantages of the breeding program.

The level of exotic inheritance for sires was set at 75% for all alternative BPs. As a result, crossbred generations will be stabilized at 75% exotic inheritance, and thus, adaptive and productive synthetic breeds will be developed in the long term. The comparison of these BPs considered predicted genetic gain, generation interval, the accuracy of selection, and discounted cost, return, and profit.

The alternative BPs were modeled using ZPLAN+ software (Täubert et al., 2011; Vit Verden, 2011). The program follows the deterministic approach that considers population mean and variance. The evaluation of the BP was based on the gene flow method and selection index procedure. That means the fraction of the genes of bulls and cows in successive generations and its associated contribution of breeding values will be considered. ZPLAN+ predicts the genetic gain for the breeding goal traits. In addition, from an economic perspective, the program calculates the discounted cost, return, and profit of BP during the investment. The program requires phenotypic and genetic parameters of breeding goal traits, economic value of traits, fixed and variable costs of selection groups, and other biological coefficients of the study population as input.

#### 2.2.1 Conventional on-station progeny testing

This BP was designed with a focus on two main populations: the dairy research herd (tier 1) maintained at the Holeta Agricultural Research Center (HARC) and the crossbred population owned by smallholder farmers in central Ethiopia (tier 2). The HARC has been actively evaluating crossbred dairy cattle and developing synthetic dairy cattle breeds for the past 50 years. However, the program faces challenges due to the small population at the on-station level and the lack of a connection with the larger crossbred population of smallholder farmers. Furthermore, crossbreeding practices among smallholder farmers have not been supported by appropriate breeding programs. Therefore, the SPT aims to bridge these two populations and assist smallholder farmers in developing more adaptive and productive crossbred cattle populations.

The SPT selects high-grade bulls (with 75% exotic inheritance) born from on-station cows at the HARC BU and uses these bulls to mate with the crossbred cattle population of smallholder farmers (the PUs) in the study areas. Table 1 presents the gene transmission matrix for the selection groups. Following the gene flow method, gene transfer occurs from the selection group in the column to the selection group in the row. Information regarding growth, production, reproduction, and survival was collected exclusively from the BU. The selection groups consist of exotic dairy breeds, zebu, and their crosses, with nine selection groups (18 paths). Exotic semen is used as the sire, while zebu cows are the dam breed for this breeding program simulation. The F1 crossbred cows are then backcrossed with an exotic sire to generate 75% dairy inheritance test bulls and cows. The sire selection process follows a two-stage selection procedure, utilizing information (performance data) from the progeny, dam, and the dam’s half-siblings (Willam et al., 2008; Vit Verden, 2011).

**TABLE 1:** Transmission matrix for the on-station bull selection program.

Genetic group	ES	ED	ZS	ZD	F1C1	HGTB	HGSB	HGCF1	CPU
ES	ES > ES	ED > ES							
ED	ES > ED	ED > ED							
ZS			ZS > ZS	ZD > ZS					
ZD			ZS > ZD	ZD > ZD					
F1C1	ES > F1C1			ZD > F1C1					
HGB	ES > HGB				F1C1>HGB				
HGCF1	ES > HGCF1				F1C1> HGCF1				
HGCF2						HGTB > HGCF2		HGCF1> HGCF2	
CPU							HGSB > CPU		CPU > CPU

ES, exotic dairy sire; ED, exotic dairy dam; ZS, zebu sire; ZD, zebu dam; F1C1, first-generation crossbred cow of exotic dairy sire and zebu dam; HGTB, high-grade test bull (crosses of ES and F1C1); HGSB, high-grade selected bull (crosses of ES and F1C1 and progeny tested); HGB, indicate logical selection group as HGSB is selected from HGTB (two-stage selection); HGCF1, high-grade cows (crosses of ES and F1C1); HGCF2, second-generation high-grade cows; CPU, cows in production unit.

The HGTB is progeny tested within the same herd by mating with HGCF1 cows to produce second-generation cows (HGCF2). Information from the dam and the dam’s half-siblings is used to evaluate the HGTB. The best high-grade bulls (HGSB) are selected from the HGTB using information from the progeny (3–4 daughters per sire), the dam, and the dam’s half-siblings. Finally, semen collected from the HGSB (progeny-tested selected bulls) is used to inseminate the crossbred cattle population owned by smallholder farmer PUs.

The number of cattle in the BU is based on the average fixed herd size at HARC over the past 5 years. Table 2 summarizes the total number of study animals in each selection group, the number of proven animals (animals with information), and the number of cows and bulls selected per year. Imported semen or semen produced by the Ethiopian Livestock Development Institute (LDI) can be used for the practical implementation of the breeding program.

**TABLE 2:** Number of bulls and cows proven and selected per year for simulation of the on-station bull selection program.

Genetic group	Number	Selected	Proven
Exotic sire (ES)	8	7	8
Exotic dam (ED)	98	13	31
Zebu sire (ZS)	8	7	8
Zebu dam (ZD)	98	13	31
First-generation exotic × zebu crossbred cows (F1C1)	100	17	33
75% exotic × 25% zebu crossbred test bull (HGTB)	20	10	20
75% exotic × 25% zebu crossbred old/selected bull (HGOB)	10	5	10
75% exotic × 25% zebu F1 crossbred cows in the breeding unit (HGCF1)	75	13	32
75% exotic × 25% zebu F2 crossbred cows (progeny of HG bull) in the breeding unit (HGCF2)	56	11	24
Cows in production unit (CPU)	143576	25456	45944

#### 2.2.2 Conventional on-farm progeny testing

This BP represents a traditional breeding scheme (progeny testing scheme based on pedigree records) commonly used in developed countries for the improvement of dairy breeds (Mulder et al., 2005; Hayes et al., 2009; Täubert et al., 2011; Mrode et al., 2019). Although there is currently no recording system in place at the smallholder level in Ethiopia, it is possible to establish a community-based recording system with the help of progressive farmers.

This breeding program has two tiers: the breeding unit (BU) and the production unit (PU). The BU includes selected crossbred cows (CBU) from the smallholder farmers’ crossbred cattle population based on breeding values of desired traits. The remaining crossbred cows in the study areas are the cows in the production unit (CPU) within the PU (Table 3). All necessary performance records and production of sires were undertaken at on-farm BUs. The number of cows in the BU was set at 40 daughters per sire for progeny testing (Taneja, 1999).

**TABLE 3:** Transmission matrix for the on-farm progeny testing scheme.

Genetic group	TBBU	SBBU	CBU	CPU
BBU	TBBU > BBU		CBU > BBU	
CBU	TBBU > CBU		CBU > CBU	
CPU		SBBU > CPU		CPU > CPU

BBU, in this alternative breeding program indicate selected bulls (SBBU) were choosen from test bullest (TBBU) which is called logical selection group (two stage selection) by the wombat software. TBBU and SBBU should have common name in the column (gene receiver).

Initially, approximately 2,500 CBU were selected from the smallholder farmers’ cattle population based on their own phenotypic records. Over time, pedigree records can be developed to select cows and bulls using estimated breeding values for desired traits. Of the 800 male calves born from the CBU (2,500 cows × 0.5 sex ratio × 0.8 conception rate × 0.8 survival rate), 500 young male calves were recruited each year, considering non-genetic factors, including physical dairy characteristics. Sire selection followed a two-stage procedure. Twenty test bulls were selected from the 500 male calves recruited based on performance data from the dam and half-siblings of the dam. These selected 20 test bulls, with 75% exotic inheritance, were purchased from farmers and raised at the station (HARC) until they reached the age for semen collection. The cows in the BU were then mated with semen collected from the test bulls for progeny testing. Of the 20 test bulls, 10 progeny-tested sires were selected based on information from the progeny, dam, and half-siblings of the dam. The genetic gain in the BU will be transferred to cows in the PU through artificial insemination using semen collected from the selected bulls. The number of cows and sires in each selection group for FPT is provided in Table 4.

**TABLE 4:** Number of bulls and cows used for the simulation of the on-farm progeny testing scheme.

Genetic group	Number	Selected	Proven
Cows in breeding unit (kept by farmers and selected for progeny testing) (CBU)	2500	2500	3125
Test bull used for progeny testing (TBBU)	500	20	500
Progeny-tested bull (SBBU)	10	10	20
Cows in production unit (CPU)	141,076	25,013	45,144

#### 2.2.3 Conventional on-station and on-farm progeny testing

This breeding program was proposed as an alternative to address the potential gap between SPT and FPT. In SPT, the number of cows available at HARC did not meet the standard required for progeny testing. Taking into account factors such as sex ratio, conception rate, and survival rate, only around 20 male calves were obtained for testing per year. Additionally, the number of available cows (24 per year) for producing sire progeny was very low. This limited number of daughters per sire (approximately 3–4) raised concerns about the accuracy of estimation. To meet the standard for progeny testing and achieve reasonable accuracy (Taneja, 1999; Archer et al., 2004; Mulder et al., 2005; Täubert et al., 2011), it was necessary to explore options to maximize the number of cows for progeny testing. At the same time, there was no established recording system at the farmer level to initiate the breeding program directly from FPT, and it would take a longer time to establish pedigree records. Additionally, determining the level of exotic inheritance of bulls for FPT required pedigree records or admixture analysis. Therefore, this breeding program combines elements of both SPT and FPT.

The simulated SFPT program was structured with three tiers, consisting of two BUs and one PU. The first BU comprised the crossbred cattle population at HARC, which served as the bull dam to produce and select bulls with 75% exotic inheritance. The number of dams at the HARC station was increased to obtain 100 young calves per year. The second tier (sub-nucleus) included 2,500 crossbred cows (CBU) selected from smallholder farmers’ cattle, forming the second BU for progeny testing purposes. Test bull production was conducted exclusively at the HARC station, while the actual progeny testing took place on-farm within the CBU. The gene transmission matrix and the number of cows and sires in each selection group for SFPT are presented in Table 5 and Table 6, respectively. Similar to SPT, this program had nine selection groups. Exotic dairy sire semen was used as the sire, while zebu cows served as the dam line. The F1 crossbred cows were backcrossed with an exotic sire to generate 75% exotic dairy inheritance test bulls. Sire selection involved a two-stage procedure. The 20 test bulls were progeny tested within the CBU, and the top 10 bulls were selected. Each progeny-tested bull was evaluated using information from the dam, half-siblings of the dam, and 40 progenies. Finally, semen collected from the selected bulls was used to inseminate the remaining crossbreds owned by smallholder farmers (CPU) in the study areas (tier 3) to transfer genetic gain. Unlike SPT, there was no on-station progeny testing within the SFPT program.

**TABLE 5:** Transmission matrix for on-station bull selection and on-farm progeny testing program.

Genetic group	ES	ED	ZS	ZD	F1C	HGTB	HGSB	CBU	CPU
ES	ES > ES	ED > ES							
ED	ES > ED	ED > ED							
ZS			ZS > ZS	ZD > ZS					
ZD			ZS > ZD	ZD > ZD					
F1C	ES > F1C			ZD > F1C					
HGB	ES > HGB				F1C > HGB				
CBU						HGTB > CBU		CBU > CBU	
CPU							HGSB > CPU		CPU > CPU

ES, exotic dairy sire; ED, exotic dairy dam; ZS, zebu sire; ZD, zebu dam; F1C, first-generation crossbred cow of exotic sire and zebu dam; HGTB, high-grade test bull (crosses of ES and F1C); HGSB, high-grade selected bull (crosses of ES and F1C and progeny tested); HGB, indicate logical selection group as HGSB is selected from HGTB (two-stage selection); CBU, cows in breeding unit (kept by farmers and selected for progeny testing); CPU, cows in production unit.

**TABLE 6:** Number of bulls and cows used to simulate on-station bull selection and on-farm progeny testing program.

Selection group	Number	Selected	Proven
Exotic sire (ES)	8	7	8
Exotic dam (ED)	98	20	31
Zebu sire (ZS)	8	7	8
Zebu dam (ZD)	200	27	64
First-generation exotic × zebu crossbred cows (F1C)	315	55	64
75% exotic × 25% zebu crossbred test bull (HGTB)	100	20	100
Cows in breeding unit (CBU)	2500	2500	3125
75% exotic × 25% zebu crossbred old/selected bull (HGSB)	10	10	20
Cows in production unit (CPU)	141,076	25,013	45,144

#### 2.2.4 Genomic selection

In the past two decades, the implementation of genomic selection has brought significant advancements in dairy genetic gain. Various studies have demonstrated that this system has effectively doubled the rate of genetic improvement in dairy traits compared to traditional progeny testing schemes (Schaeffer, 2006; Hayes et al., 2009; Garcia et al., 2016). Genomic selection involves the selection of bulls based on their genomic breeding values (GEBV), which are estimated using single nucleotide polymorphisms (SNPs). The effect of each SNP is determined through analysis of a reference population, where animals are genotyped, and their phenotype information is collected (Hayes et al., 2009; Al Kalaldeh et al., 2021). The genomic selection program offers three main advantages: 1) it enhances the accuracy of estimation by avoiding errors that can occur in pedigree records for the relationship matrix, 2) it reduces the generation interval as bulls can be selected at a younger age, and 3) it lowers the cost of the breeding program by eliminating expenses associated with progeny testing. Therefore, genomic selection was simulated to harness the potential of genomic technology for dairy development in Ethiopia.

The simulated GS program consists of two tiers with three selection groups (six paths). Tier 1 includes genotyped bulls (BBU) and genotyped cows in the reference population (CBU), while tier 2 comprises the cows in the production unit (CPU), representing the remaining crossbred cow population in the study areas. Approximately 2,500 cows (CBU) were selected from smallholder farmers’ cattle based on their own phenotypic records and then genotyped to establish the reference population (breeding unit). Information from the genotyped population, along with their phenotypic records, was used to estimate the allelic effects of SNPs and select bull dams. Each year, around 500 young male calves born from CBU were recruited, utilizing non-genetic information collected from the dam and half-siblings of the dam. From the pool of 500 genotyped male calves, 20 bulls (BBU) were selected based on their GEBV. These top 20 bulls (BBU) were purchased from farmers and raised at HARC or LDI until they reached the age for semen collection. The semen collected from these bulls (with 75% exotic inheritance) was used to inseminate cows in the breeding and production units. In the GS scheme, there was no progeny testing or on-station cow production. The gene flow matrix and the number of animals in each selection group for GS are indicated in Table 7 and Table 8, respectively.

**TABLE 7:** Transmission matrix for the genomic selection program without progeny testing.

Genetic group	BBU	CBU	CPU
BBU	BBU > BBU	CBU > BBU	
CBU	BBU > CBU	CBU > CBU	
CPU	BBU > CPU		CPU > CPU

BBU, genotyped high-grade bull; CBU, cows in breeding unit (kept by farmers and selected for reference population); CPU, cows in production unit.

**TABLE 8:** Number of bulls and cows used for simulation of the genomic selection program practiced without progeny testing.

Selection group	Number	Selected	Proven
Genotyped cows used as reference population (CBU)	2500	2500	3125
Genotyped bull (BBU)	500	20	500
Cows in production unit (CPU)	141,076	25,013	45,144

#### 2.2.5 Genomic progeny testing

Several studies have indicated that combining GS with progeny testing can significantly improve the accuracy of selection (Hayes et al., 2009; König et al., 2009; Täubert et al., 2011). Building upon this knowledge, the GPT approach was developed as an additional alternative. The GPT combines GS and progeny testing, where bull selection is conducted through a two-stage selection process. The key distinction from GS is the inclusion of progeny testing. The assumptions underlying GPT are as follows: Similar to GS, a reference population (breeding unit) consisting of 2,500 cows (CBU) was formed by selecting cattle from smallholder farmers based on their own phenotypic records. These cows were then genotyped to establish the reference population. Each year, 500 young male calves born from CBU were recruited, incorporating non-genetic information obtained from the dam and half-siblings of the dam. Of the 500 genotyped male calves, 20 test bulls were selected based on their genomic estimated breeding values (GEBV). These 20 test bulls, with 75% exotic inheritance, were purchased from farmers and raised at a station until they reached the age for semen collection.

The cows in the reference population (CBU) were inseminated with semen collected from test bulls for progeny testing. Subsequently, of the 20 test bulls, the 10 best sires were selected based on their progeny performance, as well as information from the dam and half-siblings of the dam. Semen collected from the selected bulls was used to inseminate the cows in the production unit. The transmission matrix and the number of animals in different selection groups align with those of the conventional on-farm progeny testing (FPT) approach (Table 3; Table 4).

### 2.3 Genetic parameters and economic value of breeding goal traits

The genetic parameters and economic values of breeding goal traits were determined for the study. Estimates for economic value, phenotypic standard deviation, correlations, heritabilities, and repeatability were obtained from survey data collected in the study areas and long-term data on dairy cattle at the HARC (Table 9; Table 10). More detailed genetic parameter information can be found in Direba et al. (2022). For genomic information, input parameters were sourced from relevant literature reports due to the absence of estimates for the target population. The accuracy of polygenic breeding values and the number of animals in the reference population for MY were taken from Erbe et al. (2012). Accuracy estimates for AFC, CI, and HL, along with associated numbers of animals in the reference population, were obtained from the studies of Boison et al. (2017) and Haile-Mariam et al. (2013).

**TABLE 9:** Economic value per unit change, phenotypic standard deviation (SDP), genetic parameters of the crossbred cattle, and genomic measures (1 USD = 39.55696 ETB).

Traits	Economic value in ETB	SDP*	h^2^	*r* ^2^	N	r (TI)
MY	13.38	830.98	0.30	0.52	1897	0.58
AFC	−16.19	188.27	0.19	-	1582	0.475
CI	−33.58	125.28	0.09	0.19	1783	0.51
HL	79.55	2.79	0.28	-	1883	0.34

MY, lactation milk yield; AFC, age at first calving; CI, calving interval; HL, herd life; *SDP, phenotypic standard deviation of lactation milk yield in kg; *SDP phenotypic standard deviation of AFC, CI, and HL, in days; h^2^, heritability; *r* ^2^, repeatability; N, number of animals in the reference population; r(TI) = accuracy of the polygenic breeding value. Proportion of genetic variance explained by markers (Q) = 0.7666 and number of independently segregating QTLs (K) = 1000, QTLs = quantitative trait loci.

Source: Direba et al., 2022; Erbe et al., 2012; Boison et al., 2017; Haile-Mariam et al., 2013.

**TABLE 10:** Genetic and phenotypic correlation of production, reproduction, and herd life traits.

Trait	Traits			
	MY	CI	AFC	HL
MY		0.64 ± 0.12	0.37 ± 0.13	0.64 ± 0.18
CI	0.23 ± 0.02		0.61 ± 0.19	0.78 ± 0.14
AFC	0.17 ± 0.04	0.02 ± 0.04		0.15 ± 0.21
HL	0.07 ± 0.00	0.14 ± 0.03	−0.01 ± 0.04	

MY, lactation milk yield; CI, calving interval; AFC, age at first calving; HL, herd life; Above diagonal, genetic correlation; below diagonal, phenotypic correlation.

Source: Direba et al., 2022.

Productive life, reproductive cycle, age at first reproduction, and survival rate for both zebu and crossbred cows with different levels of exotic inheritance were also derived from long-term data collected at HARC (Direba et al., 2022). Notably, zebu cows exhibited a longer productive life (7.44 years) than crossbred cows, which could be attributed to the adaptive qualities of indigenous breeds and the specific objectives of the farming systems. Differences in reproductive cycles were observed among the selection groups, with the F1 generation displaying a shorter cycle (1.26 years) than other groups. This indicates performance variations within the different selection groups (Table 11). Biological parameters based on 75% exotic inheritance were applied to the CBU and CPU, as admixture analysis revealed a similar level of exotic inheritance (78%–79%) for cows managed by smallholder farmers (Strucken et al., 2017; Netsanet et al., 2021).

**TABLE 11:** Biological coefficients used for breeding programs.

Input parameter	Unit	Value	Input parameter	Unit	Value
Productive life of test bull	Year	1	Reproductive cycle of F1 cows	Year	1.26
Productive life of high-grade cows, CBU, and CPU	Year	5.64	Reproductive cycle of CBU genotyped bulls and CPU	Year	1.32
Productive life of F1 cows	Year	5.77	Reproductive cycle of high-grade cows and bulls raised at on-station	Year	1.42
Productive life old/selected bull	Year	2	Reproductive cycle of Zebu and Friesian sire and dam	Year	1.44
Productive life of Friesian cows	Year	3.5	Sex ratio	%	0.5
Productive life of Zebu cows	Year	7.44	Conception rate	%	0.8
Age at first reproduction of Friesian and F1 cows	Year	3.15	Survival rate	%	0.8
Age at first reproduction of high-grade cows, CBU, and CPU	Year	3.52	Interest rate to calculate discounted cost and return	%	0.095
Age at first reproduction of Zebu cows	Year	3.57	Investment duration	Year	25

CBU, cows in breeding unit; CPU, cows in production unit.

Source: Direba et al., 2022.

### 2.4 Variable and fixed costs

Table 12 provides a detailed breakdown of the annual variable and fixed costs per animal for the simulated alternative BPs. The study assumed a 25-year investment period, with a 9.5% interest rate applied to both costs and returns. It is important to note that all the costs, returns, and profits estimated in this study were specifically related to the additional expenses incurred and profits obtained as a direct consequence of implementing the breeding program (Nitter et al*.,* 1994; Tadele et al*.,* 2011; Kahsa et al., 2012). Fixed costs were defined as constant expenses related to salaries, overhead costs, and computers. These costs encompassed the purchase of computers, overhead expenses (including training and monitoring costs), and salaries for labor, veterinarians, recorders, artificial insemination (AI) technicians, and animal breeders. Variable costs represented the total expenses incurred for selecting, testing, and choosing animals for the breeding program (Täubert et al., 2011; Vit Verden, 2011). Additional costs for items such as feed, genotyping, bull purchases, ear tags, veterinary services, and other administrative expenses that arose due to the BP were considered variable costs.

**TABLE 12:** Costs of inputs considered per animal per year in ETB (1 USD = 39.55696 ETB).

Input	SPT	FPT	SFPT	GS	GPT
Cost of feed/cows (to on-station breeding unit)	29,200	-	29,200	-	-
Cost of feed/bulls	18,250	18,250	18,250	18,250	18,250
Cost of genotyping/bull	-	-	-	1437.26	1437.26
Cost of genotyping/cow	-	-		339.78	339.78
Cost of purchasing genotyped bull	-			27,150	27,150
Cost of purchasing selected bull	-	22625	-	-	-
Cost of animal health/cow	106.06	-	106.06	-	-
Cost of animal health/bull	106.06	106.06	106.06	106.06	106.06
AI cost/cow	410	94.06	*410 + 94.04	94.04	94.04
Cost of ear tag/cow	100	100	100	100	100
Cost of salary/wages/cow	3624.31	1157.99	1123.54	1157.99	1157.99
Overhead cost (training, supervision, and other administration)/animal	1170.96	400	321.23	400	400
Variable cost/cow	29,816.11	194.04	*29716.11 + 194.04	533.82	533.82
Variable cost/sire	18,456.11	41,081.06	18,356.11	47,043.32	47,043.32
Fixed cost/animal	4818.69	1575.99	1459.23	1575.99	1575.99
Total annual cost required for BU	15,154,059	5,009,597	22,073,307	6,668,175	6,668,175

SPT, conventional on-station progeny testing; FPT, conventional on-farm progeny testing; SFPT, conventional on-station and on-farm progeny testing; GS, genomic selection; GPT, genomic progeny testing; *, cost for on-station + on-farm; BU, breeding unit.

The costs associated with the BPs varied depending on the specific scenarios. For example, in the case of conventional on-SPT and conventional on-SFPT, the cost of feed for cows was included because these cows were entirely used for the breeding program. However, in other BPs, where cows were part of normal dairy business processes, no additional feed costs were considered. The cost of feed for bulls was included in all BPs because bulls were recruited from the on-station breeding unit or purchased from on-farm breeding units and managed at the station until sufficient semen was collected.

In terms of genotyping, the cost for genotyping cows and bulls was 1,916.35 ETB per animal (1 USD = 39.55696 ETB) for GS and GPT. Human resources required for data collection, animal health management, supervision, and data analysis were also factored into the costs. For BPs with on-farm data gathering components (FPT, GS, and GPT), the cost included 25 data recorders (6,500 ETB per person per month) and three animal breeders (16,437 ETB per person per month). In the simulation of SPT, the cost included 20 daily laborers (1,500 ETB per person per month), two data recorders (1,890 ETB per person per month), and three animal breeders (16,437 ETB per person per month). The cost of veterinary services per animal was 94.04 ETB per year for all BPs. Additionally, the salaries of two veterinarians (16,437 ETB per person per month) and two AI technicians (6,500 ETB per person per month) were assumed for the analysis of SPT and SFPT, as these programs required full-time animal health management and AI services.

Approximately 500,000 ETB per year were assumed for training, monitoring, and other administration costs for SPT. This cost was escalated to 1,000,000 ETB per year for each of the remaining BPs, as more supervision was required to mobilize farmers and provide training for data recorders and farmers participating in on-farm breeding units. However, only the cost of AI service was included for CPU because the genetic transfer was through AI, and all farmers should use AI for the practical implementation of BP.

Costs related to the price of animals, animal health services, and AI services were obtained from the survey conducted with smallholder farmers (Direba et al*.,* 2020). The price to purchase bulls/male calves was enhanced by 25% for non-genotyped and 50% for genotyped bulls/male calves to provide better market value for genetically merited bulls and encourage farmers to sell selected young bull/male calves to the BP. Estimates of daily labor and salaries were taken from the HARC payment standard. Feed requirements and associated costs were derived from the HARC feeding standards and market costs of 2021.

## 3 Results

### 3.1 Genetic gain

The results in Table 13 show estimates of genetic gain (GG) of breeding goal traits per year and per generation. The GGs predicted for lactation MY per year were 34.52 kg, 49.63 kg, 29.35 kg, 76.16 kg, and 77.51 kg for SPT, FPT, SFPT, GS, and GPT, respectively. The corresponding GGs per generation were 209.27 kg for SPT, 273.22 kg for FPT, 174.30 kg for SFPT, 362.33 for GS, and 426.71 for GPT. The genomic BPs attain at least 26.53 kg more GG per year than the conventional BPs.

**TABLE 13:** Estimated genetic gain per year (per generation in bracket) for breeding programs.

Trait	SPT	FPT	SFPT	GS	GPT
MY (kg)	34.52 (209.27)	49.63 (273.22)	29.35 (174.30)	76.16 (362.33)	77.51 (426.71)
AFC (day)	1.19 (7.22)	0.97 (5.37)	0.69 (4.12)	1.11 (5.26)	0.90 (4.94)
CI (day)	1.57 (9.49)	2.06 (11.34)	1.20 (7.13)	2.35 (11.18)	2.29 (12.63)
HL (day)	0.07 (0.44)	0.11 (0.59)	0.06 (0.36)	0.12 (0.55)	0.12 (0.65)

MY, milk yield; AFC, age at first calving; CI, calving interval; HL, herd life; SPT, conventional on-station progeny testing; FPT, conventional on-farm progeny testing; SFPT, conventional on-station and on-farm progeny testing; GS, genomic selection; GPT, genomic progeny testing.

The GGs calculated for age at first calving (AFC) were 1.19 and 7.22 days for SPT, 0.97 and 5.37 days for FPT, 0.69 and 4.12 days for SFPT, 1.11 and 5.26 days for GS, and 0.90 and 4.94 days for GPT per year and generation, respectively. A lower GG of calving interval (CI) was recorded in SFPT (1.20 days per year and 7.13 days per generation) than other BPs (varied from 1.57 to 2.35 days per year). The value of GG obtained for CI was almost similar among FPT, GS, and GPT. The estimates of GG for herd life (HL) were very low (0.06–0.12 days per year) and almost similar among all alternative BPs.

### 3.2 Generation interval and accuracy

Generation interval (GI) refers to the age of parents when their replacement offspring is born. When the GI is low, the transfer of genetic gain from generation to generation becomes rapid. Hence, there will be higher return and genetic progress in breeding goal traits for the target population. The values of predicted GI for conventional BPs were close to each other and ranged from 5.51 years to 6.06 years (Table 14). However, GI was reduced by 21% in GS compared to SPT and reduced by 19% compared to SFPT. Table 14 summarizes the accuracy of selection for test and progeny-tested bull in different BPs. The accuracy of selection calculated for test bulls was 0.33 for SPT, 0.34 for FPT, 0.37 for SFPT, and 0.43 for both genomic BPs. Furthermore, the accuracies of the selection of progeny-tested bull estimated here were 0.85 for SPT and 0.99 for other BPs.

**TABLE 14:** Accuracy of selection index and generation interval for the alternative breeding program.

Breeding program	Accuracy of selection index		Generation interval
	Test bull	Selected bull	
SPT	0.33	0.85	6.06
FPT	0.34	0.99	5.51
SFPT	0.37	0.99	5.94
GS	0.43		4.76
GPT	0.43	0.99	5.51

SPT, conventional on-station progeny testing; FPT, conventional on-farm progeny testing; SFPT, conventional on-station and on-farm progeny testing; GS, genomic selection; GPT, genomic progeny testing.

### 3.3 Discounted profit of alternative breeding programs

The ultimate goal of the BP is to ensure the profitability and sustainability of the dairy business for the target population. As indicated in Table 12, the total costs calculated for breeding units varied among simulated BPs. The annual cost for BUs in ETB was approximately 15.15 million for SPT, 5.00 million for FPT, 22.07 million for SFPT, and 6.67 million for each genomic BP. ZLAN+ distributes costs and returns for the entire cow population included in the BP as the genetic gain obtained at the breeding unit is transmitted to the whole population (Täubert et al., 2011; Vit Verden, 2011). Furthermore, the program discounted the cost of BP considering the interest rate, generation interval, and number of animals in different selection groups.

Table 15 contains discounted cost, return, and profit per animal per generation and year. The discounted costs of BPs per animal per generation in ETB were 45.85, 52.71, 71.51, 190.28, and 172.57 for SPT, FPT, SFPT, GS, and GPT, respectively. The corresponding discounted returns were ETB 1,559.07 for SPT, 2,475.83 for FPT, 1,765.54 for SFPT, 5,741.69 for GS, and 3,969.88 for GPT. The discounted profit of the BPs ranged from ETB 1,513.22 in SPT to 5,551.40 in GS per cow per generation.

**TABLE 15:** Discounted cost, return, and profit per animal in ETB (1 USD = 39.55696 ETB).

Parameter	SPT	FPT	SFPT	GS	GPT
Cost per generation	45.85	52.71	71.51	190.28	172.57
Return per year	257.15	449.74	297.32	1206.91	721.14
Return per generation	1559.07	2475.83	1765.54	5741.69	3969.88
Profit per year	249.58	422.00	285.28	1166.92	689.79
Profit per generation	1513.22	2323.12	1694.03	5551.40	3797.30

SPT, conventional on-station progeny testing; FPT, conventional on-farm progeny testing; SFPT, conventional on-station and on-farm progeny testing; GS, genomic selection; GPT, genomic progeny testing.

## 4 Discussion

### 4.1 Genetic gain

Among the trait goals, milk yield had the highest GG in all BPs. This could be attributed to the availability of higher genetic variance of this trait in the study population and better heritability than other traits. Furthermore, the higher GG of MY recorded in this study could create a good opportunity for the future sustainability of BPs as MY accounts for 69% of the relative economic value of the dairy business in the study area. The result revealed that GG was positive for all BPs. However, the positive GG obtained for AFC and CI is undesirable. The overall results indicated that the application of any of the five BP could bring genetic progress in the crossbred population of the study area.

Genetic gain for MY estimated in the present study concurred with the report of other studies on different dairy breeds (Börner and Reinsch, 2012; García et al., 2016; Fedorovych et al., 2021). Opoola et al. (2020) and Tobias et al. (2010) predicted 245 kg–734 kg and 366 kg–410 kg MY GG per generation by modeling different BPs for dairy cattle in South Africa, Zimbabwe, and Kenya, respectively. Kudinov et al. (2018) calculated 56 kg–59 kg MY GG per year for black and white dairy cattle in Russia. However, a higher estimate (512 kg per generation) was reported by Mulder et al. (2005) using genetic evaluation data collected from different breeds.

Further comparison among BPs indicated that genomic systems noticeably increase the GG of MY. A better GG was obtained by GPT and GS than by conventional BPs. The results imply that the genetic progress of MY can be enhanced by 53%–164% by supporting the BP with genomic information. Scott et al. (2021) estimated that the rate of genetic gain increased by about 160% in Holsteins and 100% in Jersey cattle when genomic information was included in the progeny testing scheme in Australia. Consistent with the present study, the substantial contribution of genomic BPs was reported by several authors (Panigrahi and Parida, 2012; Thomasen et al., 2014; Boisonn et al., 2017).

Values calculated for AFC and CI are lower than the report of Opoola et al. (2020), who found 41–65 days for AFC and 20–42 days for CI GG per generation using data from Zimbabwe and South Africa. Similarly, Tobias et al. (2010) noted higher genetic response (90–101 days per generation) for AFC in Kenya than our estimate. Even though it was low, the GG obtained for AFC and CI is undesirable, as the economic value of these traits was negative when their GG became positive. Furthermore, selection only for MY may adversely prolong CI and AFC as the genetic correlation of MY with these two traits is positive. Consistent with our findings, García et al. (2016) calculated a GG of 0.03–0.88 days for HL per year for Holstein cows in the United States. It can be noted that the variation observed among BPs for AFC, CI, and HL looks insignificant. Although the GGs of AFC and CI were low, care should be taken in the selection index to further reduce the GG of these two traits.

### 4.2 Generation interval and accuracy

Genomic selection (GS) reduces the generation interval (GI) by up to 21% compared to conventional BPs. This is mainly because bulls in GS were selected and transferred genetic merit at an early age. Estimates of GI in the present study were higher than those of other studies in developed countries. Täubert et al. (2011) calculated 5.02, 3.44, and 4.64 years for conventional, genomic, and combined conventional-genomic breeding programs, respectively. Similarly, Garcia et al. (2016) discussed a reduction of GI from 7 years to 2.5 years by implementing genomic selection in dairy cattle in the United States. The difference with the current result might be due to the lack of application of appropriate genetic improvement programs in Ethiopia and farming practices, as the herd life of dairy cows was longer in the Ethiopian condition.

The accuracy of selection calculated for test bulls is comparable to the report of Brown et al. (2016), who found 0.28 to 0.41 prediction accuracy for the selection of crossbred dairy cattle in East Africa. However, Täubert et al. (2011) found better accuracy (0.54) for test bulls in Germany than the present study in conventional BPs. The improvement of accuracy from 0.3 to the 0.43 obtained in the present study is attributed to the increase in number of cows (2500) in the BU or the reference population. The result also indicated that genotyping bulls could enhance the accuracy of the selection of test bulls by 16%–30%. Similar conclusions are reported by other authors (Haile-Mariam et al., 2013; Garcia et al., 2016; Aliloo et al., 2018). The values of accuracy of selection of progeny-tested bulls in this study (0.85–0.99) are close to the 0.89 estimated for Holstein cattle in Germany (Täubert et al., 2011). Hayes et al. (2007) also reported a 0.84 accuracy using marker-assisted (haplotype) selection with phenotype records for cattle in Australia.

### 4.3 Discounted profit of alternative breeding programs

The discounted cost in genomic BPs looks higher than that of conventional systems, but it was offset by the high return in these systems. Among conventional progeny testing schemes, FPT generated 53% more profit than SPT and 37% more than SFPT. The genomic systems enhanced the profit by at least 63% compared to the conventional counterparts. The GS outperformed the SPT, SFPT, and FPT by 266%, 227%, and 138%, respectively. Similarly, the profit obtained from GPT was better than that of all conventional BPs. On the other hand, the profitability of GS was reduced by 31% when it was supported by progeny testing. The substantial difference in profit observed among BPs is attributed to the BP scenario. The costs of SPT and SFPT were inflated mainly due to the cost of animal feed and salary, as these BP maintained many cows at the station. The other BPs (FPT, GS, and GPT) kept about 20 sires at the station, which significantly reduced the cost required for animal feed and labor. However, considering the contribution of the BPs to the entire population, the calculated cost looks reasonable.

The higher profitability of genomic systems probably resulted from lower feed and labor costs for animal management and a lower generation interval, which leads to rapid genetic gain. The contribution of progeny testing BPs and further enhancement by genomic BPs for better profitability of the dairy business was inferred in several reports (Börner and Reinsch, 2012; Weller et al., 2017; Mrode et al., 2019; Newton and Berry, 2020; Scott et al., 2021). For instance, discounted profits of 18–26 USD per generation in Kenya (Kahi and Nitter, 2002), 29.92 euros per year for Danish Jersey dairy cattle (Thomasen et al., 2014), and 238 to 532 euros per generation for Holstein dairy cattle in Germany (Täubert et al., 2011) were reported for different BPs. The difference in profit and values of other parameters of the present result with other reports could be due to variations in economic values and the number of traits included in BPs, the size of the population included in the BPs, and parameters fitted in the model.

### 4.4 Comparative advantages and limitations of alternative breeding programs

All suggested BPs showed a positive profit. However, each has its own advantages and limitations. The SPT is highly suitable for record keeping and selection of animals as the BU is maintained at the station. However, the number of cows in the BU was very low to produce and recruit test bulls. This highlighted that sires were evaluated with a very low number of daughters (3–4 daughters per sire). As a result, the selection accuracy of test bulls and selected bulls was lower than other BPs. On the other hand, the annual total cost of SPT was also higher than FPT and genomic BPs.

The result showed that the GG and profit obtained by FPT were better than SPT and SFPT. Likewise, the accuracy of sire selection was better than SPT and comparable with SFPT. This BP was superior to SPT and SFPT by 53% and 37% discounted profit, respectively. Furthermore, farmers practicing FPT participate in a community that can enhance the success of a BP. The practical limitations of this BP are 1) lack of a record-keeping system at the farmer level and establishing pedigree record may take longer; 2) in the absence of a pedigree record, selection based on phenotypic performance may not bring genetic progress; and 3) it was difficult to estimate the level of exotic inheritance for sires as there were no pedigree records at the farmer level.

The combined on-SFPT could solve the shortcomings of SPT and FPT. The on-station part supports the BP by a pedigree record, and the on-farm part avails a sufficient number of cows for progeny testing. Community participation in this BP could also create a smooth environment (enhance cooperation of farmers) for practical implementation of this BP as farmers provide cows for progeny testing and support recording and benefit from the BP. Increasing the number of cows for progeny testing by participating dairy farmers has improved the accuracy of test bull selection from 0.33 to 0.37 and selected bulls from 0.85 to 0.99. The profit was increased by 11% compared to SPT. However, it was decreased by 37% compared to FPT. The GIs of SPT and SFPT were greater than all BPs. In addition, the annual cost of SFPT was also much higher than all other BPs.

As expected, the potential advantages of genomic systems were enormous. This system overtakes the conventional counterpart by at least 63% profit in GPT and 138% when GS was compared. The annual cost of genomic BPs was decreased by about 56%–69% compared to SPT and SFPT. The GI was reduced by up to 21% by using genomic BPs. Furthermore, the accuracy of test bull selection was improved to 0.43 in genomic BPs. The practical challenge of genomic BPs could be the establishment, phenotype data collection, and genotyping of the reference population.

In conclusion, all alternate BPs produced a profit and contributed to MY’s genetic gain. These imply that implementing any of the alternative BPs can bring genetic progress. Genomic BPs overtake the conventional BPs in terms of genetic gain, generation interval, accuracy of sire selection, and discounted profit. Hence, establishing GS and gradually supporting it with progeny testing (GPT) are recommended in that order as the main way forward to attain better genetic progress in dairy farms in Ethiopia and similar scenarios in other tropical countries. However, until compulsory conditions are in place for genome selection, SFPT can be considered more practical. For the success of the breeding program, relevant governmental and non-governmental institutes should be engaged, and their roles and responsibilities in the implementation of the breeding program should be defined. It is also crucial to enhance community participation through training and supervision, as well as build the capacity of a biotechnology laboratory to facilitate the genotyping of the reference population and test bulls.

## Data Availability

The datasets presented in this article are not readily available because the long-term data used for the analysis of genetic parameters are owned by the Ethiopian Institute of Agricultural Research. Requests to access the datasets should be directed to ffeyissa@yahoo.com.
